# Contribution of *in Vivo* Experimental Challenges to Understanding Flat Oyster *Ostrea edulis* Resistance to *Bonamia ostreae*

**DOI:** 10.3389/fcimb.2017.00433

**Published:** 2017-10-06

**Authors:** Benjamin Morga, Tristan Renault, Nicole Faury, Sophie Lerond, Céline Garcia, Bruno Chollet, Jean-Pierre Joly, Sylvie Lapègue, Estelle Harrang, Isabelle Arzul

**Affiliations:** ^1^Laboratoire de Génétique et Pathologie des Mollusques Marins, IFREMER Institut Français de Recherche pour l'Exploitation de la Mer, La Tremblade, France; ^2^Département Ressources Biologiques et Environnement, IFREMER Institut Français de Recherche pour l'Exploitation de la Mer, Nantes, France

**Keywords:** *Bonamia ostreae*, protozoan, *Ostrea edulis*, resistance, experimental infection, haemocytes, gene expression, apoptosis

## Abstract

Bonamiosis due to the parasite *Bonamia ostreae* has been associated with massive mortality outbreaks in European flat oyster stocks in Europe. As eradication and treatment are not possible, the control of the disease mainly relies on transfer restriction. Moreover, selection has been applied to produce resistant flat oyster families, which present better survival and lower prevalence than non-selected oysters. In order to better understand the mechanisms involved in resistance to bonamiosis, cellular and molecular responses of 2 oyster groups (selected oysters and wild-type oysters) were analyzed in the context of experimental injection and cohabitation infections. Cellular responses including non-specific esterases detection, ROS production and phagocytosis activity were analyzed by flow cytometry. Four genes homologous to those shown to be involved in immunity were selected (Inhibitor of apotosis OeIAP, Fas ligand OeFas-ligand, Oe-SOD, and OeEc-SOD) and monitored by quantitative reverse-transcription PCR (qRT-PCR). Infected oysters showed higher phagocytosis activity than controls. Infected selected oyster show a lower phagocytosis activity which might be a protection against the parasite infection. The expression of OeIAP and OeFas-ligand gene was significantly increased in selected oysters at 5 days post-injection. OeIAP gene expression appeared to be significantly increased in wild-type oysters at 8 days post-injection. Our results suggest that resistance to bonamiosis partly relies on the ability of the oysters to modulate apoptosis.

## Introduction

The European flat oyster *Ostrea edulis* is an endemic species to European and North African coasts and is found from Norway to Morocco as well as in the whole Mediterranean and Black seas (Jaziri, 1985). European production of *O. edulis* has shown a drastic decline from about 30,000 t in 1961 to less than 2,000 t nowadays in Europe (Food and Agriculture Organization of the United Nations, 2010). This decrease is mainly explained by the rapid spread and high impact of two parasitic diseases, due to *Marteilia refringens* and *Bonamia ostreae*. The parasite *Bonamia ostreae* was first described in 1979 by Pichot et al. (1979), in the context of spat mortality event in French Brittany. *Bonamia ostreae* is an intracellular rhizarian protozoan affiliated to the phylum Haplosporidia (Arzul and Carnegie, 2015; Sierra et al., 2016).

The disease has been associated with haemocyte infiltration in the connective tissue of the gills, mantle and digestive gland in relation with the multiplication of *B. ostreae* (Balouet et al., 1983; Cochennec-Laureau et al., 2003). Mortalities are mainly observed in two-year old oysters (Culloty and Mulcahy, 1996) although younger individuals (0–1 year more) and larvae have been shown susceptible to the infection (Cavalier-Smith and Chao, 2003; Lynch et al., 2005; Lallias et al., 2008; Arzul et al., 2011).

Although methods for *in vitro* cultivation of the parasite are lacking, a protocol for parasite purification from infected oysters (Mialhe et al., 1988) enables *in vitro* and *in vivo* studies on interactions between *B. ostreae* and its host cells, the haemocytes (Mourton et al., 1992; Morga et al., 2009, 2010, 2011a,b, 2012). This protocol has notably allowed the implementation of experimental infection trials by injecting the parasite into flat oysters (Mialhe et al., 1988; Hervio et al., 1995) or by cohabiting source oysters injected with a known number of *B. ostreae* cells and healthy oysters (Culloty et al., 1999; Lallias et al., 2008).

Selective breeding programs were initiated in France and Ireland in the 90's, with the main objective of producing flat oysters more resistant to bonamiosis (Naciri-Graven et al., 1998; Culloty et al., 2004). In France, genetic selection was applied through inoculation tests and field testing, the surviving oysters being used as breeders to produce the next generation (Naciri-Graven et al., 1998). As a result, different selected families have been produced in Ifremer facilities (La Tremblade, Charente-Maritime, France) and showed enhanced survival and lower prevalence of the parasite compared with control wild-type oysters in *B. ostreae* contaminated areas (Lapègue et al., 2004).

The flat oyster *Ostrea edulis* and its parasite *Bonamia ostreae* represent a well-suited model to study host-pathogen interactions. Previous studies demonstrated that the parasite *B. ostreae* was internalized after 30 min of contact with haemocytes and was not degraded after phagocytosis (Chagot et al., 1992; Mourton et al., 1992). Hervio et al. (1991), reported that the parasite possesses catalytic enzymes and acid phosphatase which could inhibit haemocyte activities. Intracellular parasites have developed sophisticated strategies to escape host defense mechanisms, thereby finding unique niches where they can survive, and from which they can establish successful infections. Recently *in vitro* infections were performed in order to study the cellular and molecular responses of haemocytes from selected and wild-type flat oyster *Ostrea edulis* against the parasite *B. ostreae* (Morga et al., 2009, 2011b). Moreover, the identification of some genes characterized by SSH contributed to better understand the immune response of *Ostrea edulis* against the intracellular parasite (Morga et al., 2012).

*In vitro* infections are widely used in order to describe the interaction between the flat oyster and its parasite *B. ostreae* (Chagot et al., 1992; Mourton et al., 1992; Morga et al., 2009, 2010; Gervais et al., 2016). However, the knowledge of the cellular and molecular responses in selected oysters is poorly studied especially in the context of *in vivo* experiments. *In vitro* infections were previously used to identify cellular and molecular mechanisms involved in the response of the haemocytes to the infection of the parasite *B. ostreae*. However, such experiments do not consider early infection steps including the interaction between mucus and parasite. On the contrary, *in vivo* infections are more similar to natural infections.

Considering the need to better understanding interactions between flat oysters and the parasite *Bonamia ostreae*, two types of experimental challenges were carried out by injecting parasites and by exposing non-infected oysters with healthy ones. Two groups of oysters were challenged in the context of these trials: flat oysters selected for their better resistance to bonamiosis and wild-type oysters collected from a bonamiosis endemic French area. These experimental infections allowed us to better describe the pathogenesis and the response of the oyster to the infection. Cellular and molecular responses of both oyster groups were analyzed by histology, and flow cytometry and by quantitative reverse-transcription PCR (qRT-PCR).

## Materials and methods

### Biological material

#### Oysters

Eighteen to twenty four months (*n* = 850) flat oysters *Ostrea edulis* were collected from Quiberon Bay (Southern Brittany, France), a bonamiosis endemic zone, and were acclimatized in Ifremer's facilities (La Tremblade, Charente Maritime, France) over 30 days. These oysters are called “wild-type oysters” thereafter.

The oysters subsequently called “selected oysters” were obtained from the progenitors identified in a previous program of selection (Naciri-Graven et al., 1998). Flat oysters were produced in the IFREMER's hatchery from Argenton (Brittany, France). Then, 18 month old oysters were acclimatized in IFREMER's facilities in La Tremblade (Charente Maritime, France) over 30 days.

#### Haemolymph collection

Haemolymph was withdrawn from the adductor muscle sinus using a 1 ml syringe equipped with a needle (0.40 mm × 90 mm). To eliminate debris the haemolymph samples were filtered through 60 μm nylon mesh and hold on ice to prevent cell aggregation. The volume of haemolymph collected from each oyster was approximately 1 ml. Haemolymph samples were pooled and haemocytes counted using a Malassez-cell.

#### Parasite purification

*Bonamia ostreae* was purified as described in Morga et al. (2009). Briefly, heavily infected oysters were selected by examination of heart tissue imprints using light microscopy. After homogenization of all the organs except the adductor muscle, parasites were concentrated by differential centrifugation on sucrose gradients and then purified by isopycnic centrifugation on a Percoll gradient. Finally, the purified B. ostreae cells were suspended in FSW and counted using a Malassez-cell.

### Experimental infections

#### Experimental infection by injection

The experimental design consisted of 12 tanks (6 for selected oysters and 6 for wild-type oysters) containing 60 oysters each. Purified parasites (2.2 × 10^5^ per individual) were injected into 180 selected oysters and 180 wild-type oysters. The same amount of oysters was injected with 100 μL of filtered sea water (FSW). Injection was performed into the adductor muscle after “anesthesia” using MgCl_2_ (200 g/l) (for injection oyster were not notched only anesthetized). Flat oysters were then maintained in 120 L tank supplied with a constant flow of seawater 150 L h^−1^ enriched in phytoplankton (*Skeletonema costatum, Isochrisis galbana, Chaetoceros gracialis* and *Tetraselmis suecica*).

**Figure 1:**
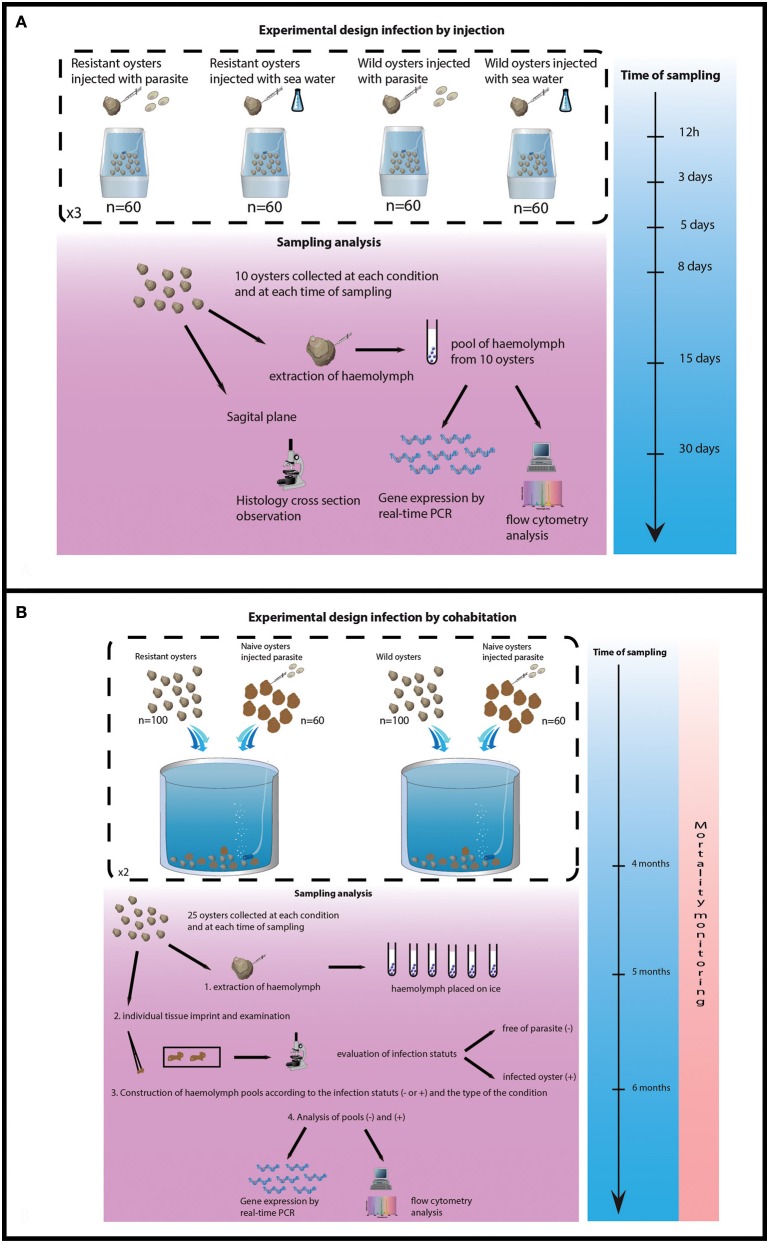
Experimental design of both trials. **(A)** Experimental design of infection by injection. **(B)** Experimental design of infection by cohabitation.

Ten oysters were collected from each tank 12 h, 3, 5, 8, 15, and 30 days after parasite injection. Haemolymph was withdrawn from the adductor muscle of each oyster and haemolymph samples (10) were pooled for each tank and each collecting date. Pools of haemolymph were analyzed by flow cytometry and real time PCR (Figure 1A).

#### Experimental infection by cohabitation

The experimental design consisted of 4 tanks, 2 tanks containing 100 selected oysters each and 2 tanks containing 100 wild-type oysters each. Cohabitation was performed between 100 challenged oysters and 60 experimentally infected oysters per tank. Oysters (selected and wild) were previously infected by injection of 1 × 10^6^ purified parasites per oyster as previously described.

The experiment lasted 6 months, oyster mortality was checked daily and dead oysters were analyzed by heart imprint. Twenty five challenged oysters were collected from each tank after 4 and 5 months of cohabitation. Remaining oysters were sacrificed at the end of the experiment (6 months post-cohabitation). Haemolymph was withdrawn from the adductor muscle of each oyster and maintained on ice. Infection status was established by heart imprint and haemolymph samples were pooled according to the type of oysters (selected/wild-type) and the status of the oysters (infected or non-infected). Pools of haemolymph were analyzed by flow cytometry and real time PCR (Figure 1B).

### Detection of *Bonamia ostreae*

#### Heart imprint

In the context of the cohabitation experiments each challenged oyster was characterized by heart imprint. Heart imprint was performed on each sampling oyster. After dissecting out, drying the ventricle on absorbent paper, several imprints were carried out on glass slides. After air drying for 2 min, the slide was then stained with Hemacolor (Merck). Slides were observed under a light microscope and the level of infection with the parasite was characterized according to Hervio et al. (1995) into the following categories: – 0 negative results (B0−) when no parasite was detected, – I low infections (B0+) when five or fewer parasites were observed, – II moderate infections (B0++) when around one parasite per microscopic field of view was detected, – III heavy infections (B0+++) when several or numerous parasites were observed in each microscopic field of view.

#### Histology

In the context of the injection experiments, for each oyster, a section of tissues including gills, mantle and digestive gland was placed in Davidson's fixative for 24 h and embedded in paraffin. Sections (5 μm) were placed in an oven at 60°C, deparaffinized in xylene for 10 min, and stained with a conventional hematoxylin and eosin staining. Intensity of infections with *Bonamia ostreae* was established as following—(I) null infection (0) when no *B. ostreae* was detected in the section (BO−); —(II) light infection when one to two *B. ostreae* were present in each infected haemocyte, rarely up to 4 (BO+); —(III) moderate infection when *B. ostreae* occurred in various foci of haemocytic infiltration and haemocytes enclosing few (1–4) parasites coexisted with haemocytes bearing up to 10 parasites (BO++); —(IV) heavy infection when *B. ostreae* was widespread throughout host organs and numerous parasites occurred in each infected haemocyte, (even more than 20) (BO+++).

### Cellular and molecular: haemocyte responses

#### Flow cytometry

Cell mortality, non-specific esterase activities, ROS (Reactive oxygen species) production and fluorescent bead phagocytosis were measured by flow cytometry using an EPICS XL 4 (Beckman Coulter) according to previously published protocols (Morga et al., 2009).

#### Quantitative reverse-transcription PCR (qRT-PCR)

Extraction of total RNA and real-time quantitative PCR reactions were performed according to previously published protocol (Morga et al., 2010, 2011a,b, 2012). The expression of tested genes was normalized using the elongation factor 1 alpha (EU651798) as housekeeping gene (Morga et al., 2010). In the context of injection, calibrator consisted of samples collected at 12 h post-infection. For cohabitation experiment, the calibrators were non-infected oysters (Bustin et al., 2009). Fold units were calculated using the method described by Pfaffl (2001) (gene expression; up-regulated >1 and down regulated <1). Genes studied in both experiment were Inhibitor of apoptosis OeIAP, Fas ligand OeFas-ligand, cytoplasmic superoxyde dismutase Oe-SOD and extracellular superoxyde dismutase OeEc-SOD.

### Statistical analysis

Flow cytometry results were analyzed using ANOVA test. Values were converted into r angular arcsinus √(% of positive cells) before analysis to ensure respect of a priori assumptions of normality and homogeneity ANOVA analyses were performed using XLSTAT-Pro® version 7.5.3 software. In the case of rejection of H0, an *a posteriori* Tukey test was used to compare differences between means. Real time PCR were analyzed using ANOVA test.

## Results

### Experimental infection by injection

#### Detection of *Bonamia ostreae*

Examination of histological sections allowed detecting *Bonamia ostreae* in challenged flat oysters. More infected oysters were reported among the wild-type group (24 positive oysters/120 analyzed oysters: 20%) than in selected oysters (6 positive oysters/120 oysters analyzed: 5%) (Table 1A). Among infected wild-type oysters, 9 showed low infection level (Bo+), 4 appeared moderately infected (Bo++) and 11 displayed high infection levels (Bo+++). Among infected selected oysters, 4 showed low infection level (Bo+) and 2 oysters were found moderately infected (Bo++) (Table 1A). While the number of infected oysters decreased in time for the selected oysters, more infected oysters were found at the end of the experiment in the wild group (Table 1B).

**Table 1:**
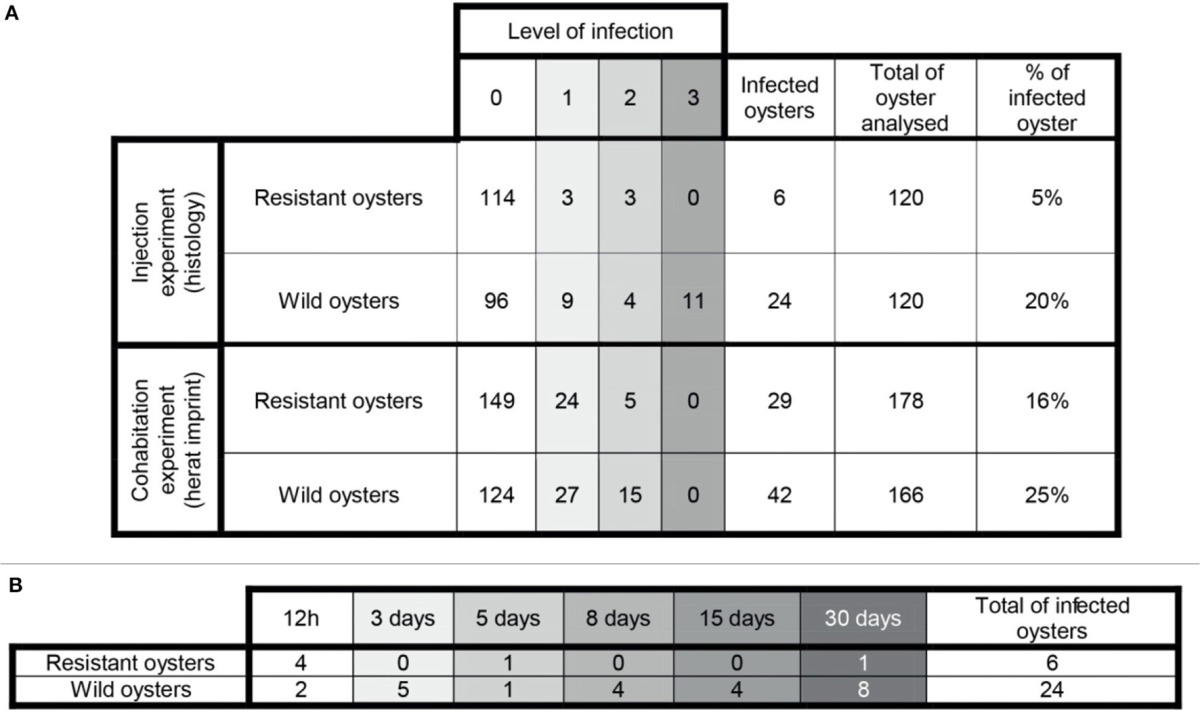
**(A)** General information about the infection level in challenged oysters. **(B)** Number of infected oysters along the experimental infection by injection.

**Figure 2:**
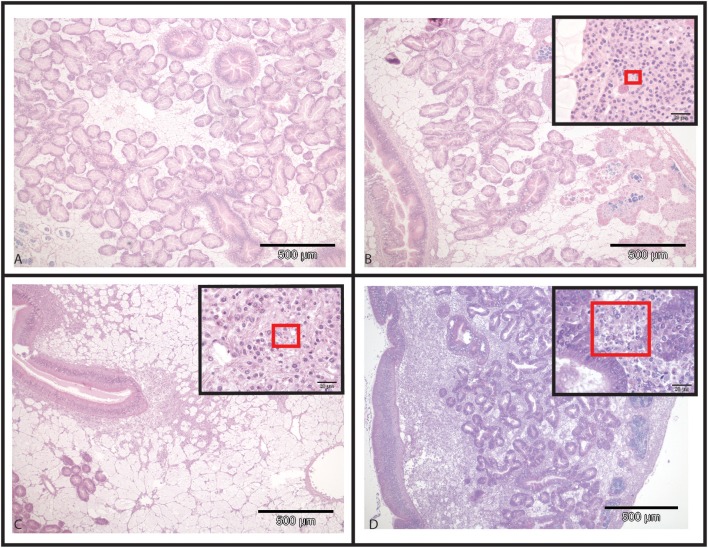
Hematoxylin and Eosin stained histological slides from flat oysters *Ostrea edulis*. **(A)** Non-infected oyster displaying normal connective tissue around the digestive tubules. **(B)** Infected oyster showing low haemocytic infiltration in the connective tissue around the digestive tubules. Higher magnification shows the presence of *Bonamia ostreae* in haemocytes contributing to this haemocytic infiltration. **(C)** Infected oyster showing moderate haemocytic infiltration in the connective tissue along the stomach. Higher magnification shows the presence of *Bonamia ostreae* in haemocytes contributing to this haemocytic infiltration. **(D)** Infected oyster showing important haemocytic infiltration in the connective tissue around digestive tubules. Higher magnification shows the presence of numerous parasites.

In infected selected oysters, infection level was generally low (few infected zones and few parasite cells observed per infected area) and the parasite was mainly detected in gills and digestive gland. On the contrary, infected wild oysters generally displayed high infection levels (with parasites widely spread in the different organs including the digestive gland, gills, mantle and gonad. Haemocyte infiltration could sometimes be observed in infected zones in wild oysters (Figure 2).

#### Flow cytometry

Haemocyte mortality rates did not present significant differences between tested conditions and never exceeded 5%.

**Figure 3:**
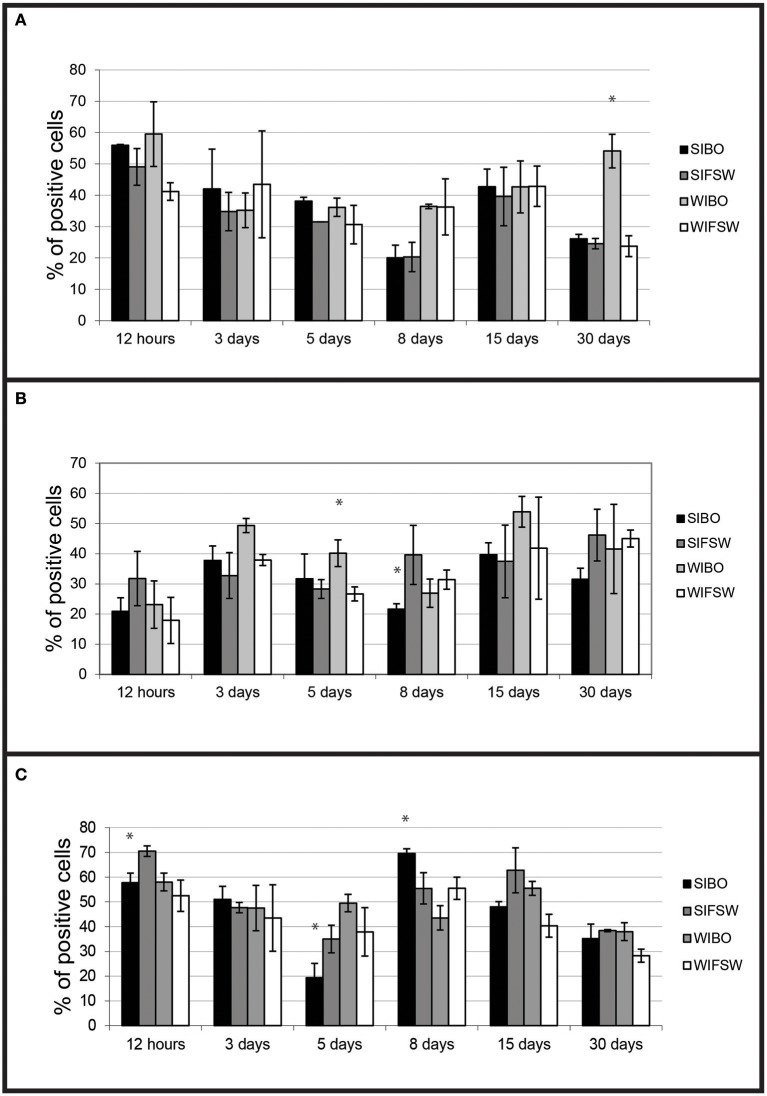
Histograms of flow cytometry (Esterase activity, ROS and phagocytosis) after an infection by injection. **(A)** Percentages of haemocytes positive for non-specific esterase activity during the experimental infection by injection. Values are means of three replicates and bars represent standard deviation. (SIBO, Selected oysters injected with Bonamia ostreae; SIFSW, Selected oysters injected with filter salt sea water; WIBO, Wild oysters injected with *Bonamia ostreae*; WIFSW, Wild oysters injected with filter salt sea water). ^*^Indicates significant difference for WIBO compared to WIFSW. **(B)** Percentages of haemocytes positive for ROS during the experimental infection by injection. Values are means of three replicates and bars represent standard deviation. (SIBO, Selected oysters injected with *Bonamia ostreae*; SIFSW, Selected oysters injected with filter salt sea water; WIBO, Wild oysters injected with *Bonamia ostreae*; WIFSW, Wild oysters injected with filter salt sea water). ^*^Indicates significant difference for WIBO compared to WIFSW or FIBO compared to SIFSW. **(C)** Percentages of haemocytes positive for phagocytosis during the experimental infection by injection. Values are means of three replicates and bars represent standard deviation. (SIBO, Selected oysters injected with *Bonamia ostreae*; SIFSW, Selected oysters injected with filter salt sea water; WIBO, Wild oysters injected with *Bonamia ostreae*; WIFSW, Wild oysters injected with filter salt sea water). ^*^Indicates significant difference for WIBO compared to WIFSW or FIBO compared to SIFSW.

Haemocytes from wild-type challenged oysters showed higher non-specific esterase activities (*p* < 0.0001) than non-infected 30 days post-injection (Figure 3A) and higher ROS production than controls 5 days (*p* < 0.016) post-injection.

Haemocytes from selected challenged flat oysters produced less ROS than controls 8 days post-injection (*p* < 0.014) (Figure 3B).

Fluorescent bead phagocytosis was lower in selected infected oysters compared with non-infected ones particularly at 12 h (*p* < 0.037) and 5 days (*p* < 0.037) post-injection. On the contrary, wild-type challenged oysters showed higher fluorescent bead phagocytosis compared with non-challenged ones at 12 h post-injection (Figure 3C).

#### Oyster gene expression

**Figure 4:**
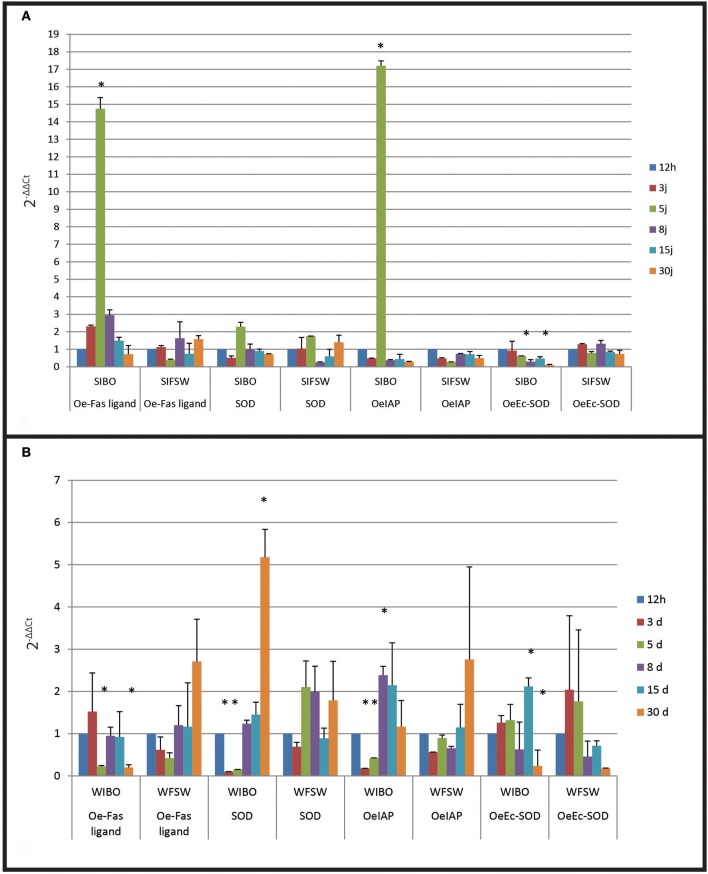
Gene expression in selected oyster and in wild oyster after infection by injection. **(A)** Relative expression by quantitative PCR of selected transcripts in resistant oyster population (OeIAP, OeFas-ligand, OeSOD, and OeEc-SOD) libraries. Expression levels were normalized to EF1-α and presented as relative expression to controls (mean ± SD, *n* = 3). ^*^Indicates significant differences of gene expression compared to controls. (SIBO: oysters injected with Bonamia ostreae, SIFSW: oysters injected with filter salt sea water). **(B)** Relative expression by quantitative PCR of selected transcripts in wild oyster population (OeIAP, OeFas-ligand, OeSOD, andOeEc-SOD) libraries. Expression levels were normalized to EF1-α and presented as relative expression to controls (mean ± SD, *n* = 3). ^*^Indicates significant differences of gene expression compared to controls. (WIBO, Wild oysters injected with Bonamia ostreae; WFSW, Wild oysters injected with filter salt sea water)

FSW (filtered sea water) injected oysters did not present significant gene expression modulation whatever the oyster group (wild or selected) was (Figures 4A,B).

**Figure 5:**
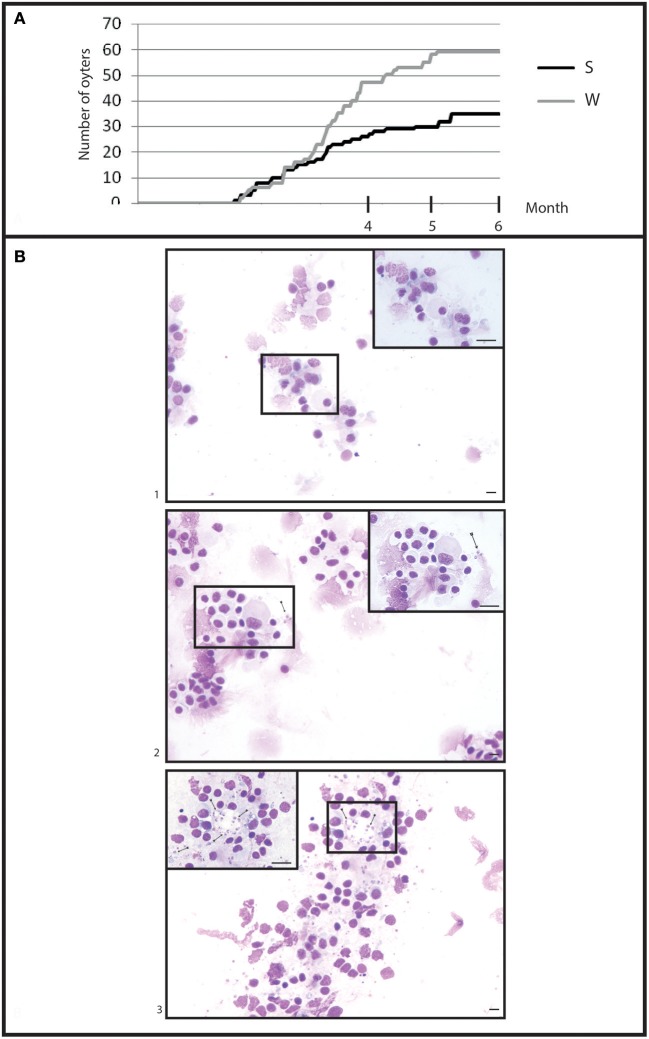
General information in infection by cohabitation. **(A)** Cumulative mortality during 6 month of cohabitation in (S) selected oyster population and (W) wild oyster population. **(B)** Heart imprint (1) low infections (B0+), (2) moderate infections (B0++), (3) heavy infections (B0+++).

Selected infected oysters showed a significant up regulation (*p* < 0.0001) of Oe-IAP and Oe-Fas ligand gene expression, especially 5 days post-injection while infected wild oysters displayed a down regulation of these genes (Figure 4). In wild-type flat oysters injected with the parasite, the expression of OeFas-ligand gene expression was significantly down regulated (*p* < 0.005) after 5 and 30 days (*p* < 0.006) (Figure 5).

SOD expression appeared down regulated at 3 days post-infection in selected infected oyster is down regulated and up regulated at 5 days in wild infected oyster expression.

In contrast, Ec SOD was down regulated at 5 days post-infection in infected selected oysters significantly up regulated at 15 days in wild oysters.

### Experimental infection by cohabitation

#### Mortality monitoring and *Bonamia ostreae* detection

Selected oysters showed a significant (*p* < 0.05) less mortality than wild-type ones (Figure 5A). At the end of the experiment, a total of 58 wild-type oysters and 35 selected oysters were found dead.

Examination of heart imprints allowed detecting *Bonamia ostreae* in some challenged flat oysters (Figure 5B). More infected oysters were reported among the wild-type group (42 positive oysters/166 oysters analyzed: 25%) than in the selected group (29 positive oysters/178 oysters analyzed: 16%) (Table 1A). Among challenged wild-type oysters 27 oysters appeared lightly infected (Bo+) and 15 oysters showed high level of infection (Bo++). Among challenged selected oysters 24 oysters were found lightly infected (Bo+) and 5 appeared highly infected significantly different to wild type oyster. According to our infection level scale, no very highly infected oyster (Bo+++) was detected whatever the oyster group was (Table 1A).

#### Flow cytometry analyses

**Figure 6:**
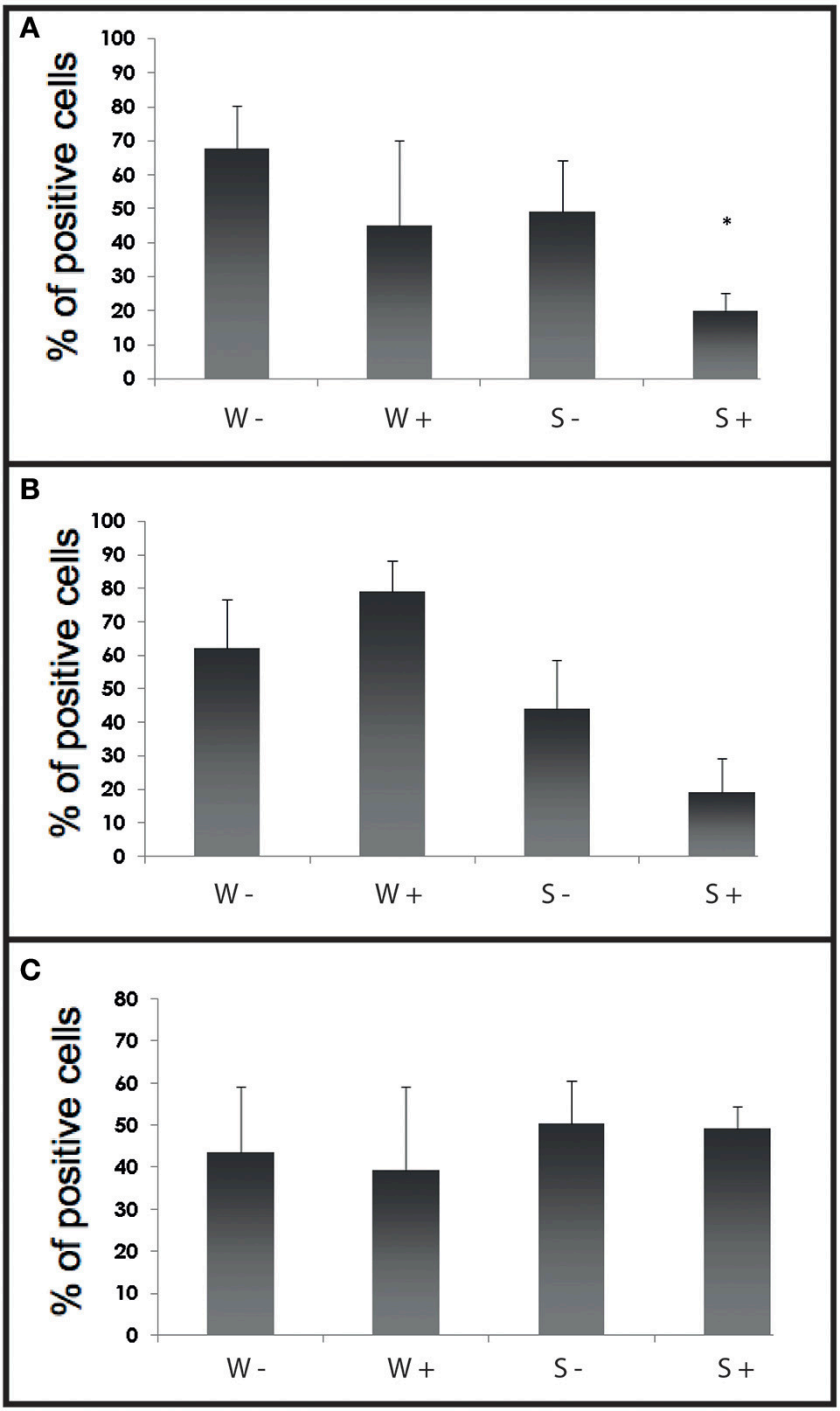
Histograms of flow cytometry (Esterase activity, ROS and phagocytosis) after an infection by cohabitation. **(A)** Percentages of haemocytes positive for phagocytosis during the experimental infection by cohabitation. Values are means of three replicates and bars represent standard deviation. W− (Wild haemolymph pool of free of parasite), W+ (wild haemolymph pool infected with the parasite *Bonamia ostreae*), S− (Selected haemolymph pool of free of parasite), S+ (selected haemolymph pool infected with the parasite *Bonamia ostreae*). **(B)** Percentages of haemocytes positive for non-specific esterase activity during the experimental infection by cohabitation. Values are means of three replicates and bars represent standard deviation. W− (Wild haemolymph pool of free of parasite), W+ (wild haemolymph pool infected with the parasite *Bonamia ostreae*), S− (Selected haemolymph pool of free of parasite), S+ (selected haemolymph pool infected with the parasite *Bonamia ostreae*). ^*^Indicates significant difference for S+ compared to S−. **(C)** Percentages of haemocytes positive for ROS during the experimental infection by cohabitation. Values are means of three replicates and bars represent standard deviation. W− (Wild haemolymph pool of free of parasite), W+ (wild haemolymph pool infected with the parasite *Bonamia ostreae*), S− (Selected haemolymph pool of free of parasite), S+ (Selected haemolymph pool infected with the parasite *Bonamia ostreae*).

Fluorescent bead phagocytosis was significantly lower in infected selected flat oysters compared to non-infected ones (*p* < 0.005, Figure 6A). Non-specific esterase activities and ROS production did not present significant difference between tested conditions (Figures 6B,C).

#### Gene expression results

**Figure 7:**
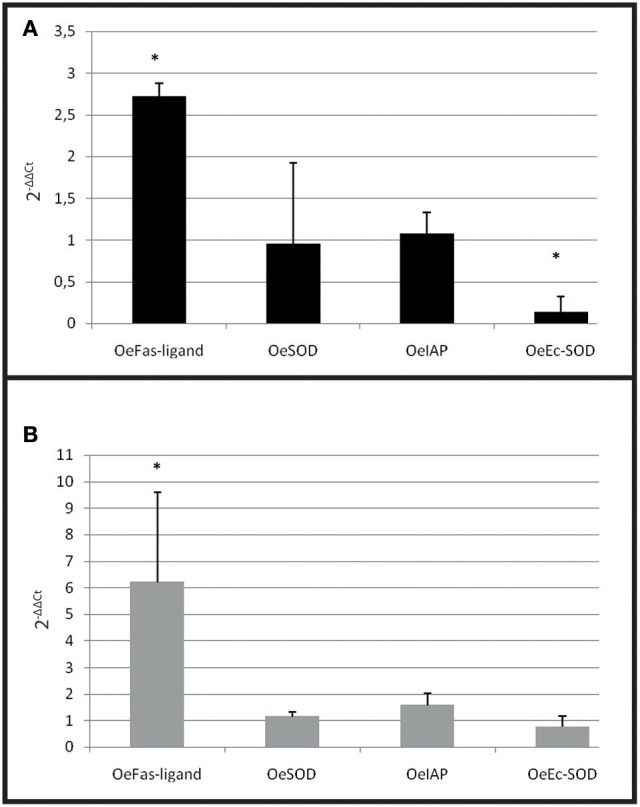
Gene expression in resistant oyster and in wild oyster after infection by cohabitation. **(A)** Relative expression by quantitative PCR of selected transcripts in resistant infected oyster population (OeIAP, OeFas-ligand, OeSOD andOeEc-SOD) libraries. Expression levels were normalized to EF1-α and presented as relative expression to controls (pool free of parasite) (mean ± SD, *n* = 2). ^*^Indicates significant differences of gene expression compared to controls. **(B)** Relative expression by quantitative PCR of selected transcripts in wild infected oyster population (OeIAP, OeFas-ligand, OeSOD, andOeEc-SOD) libraries. Expression levels were normalized to EF1-α and presented as relative expression to controls (pool free of parasite) (mean ± SD, *n* = 2). ^*^Indicates significant differences of gene expression compared to controls.

A significant over expression of Oe-Fas ligand gene (*p* < 0.0001) was observed in infected selected and wild-type oysters (Figures 7A,B). The expression of *Oe-EcSOD* gene was significantly down regulated in infected selected oysters (*p* < 0.0001) (Figure 7A).

## Discussion

Two types of experimental infections (injection of parasites and cohabitation with experimentally infected oysters) have been performed in the present study in order to investigate *in vivo the* responses of two groups of flat oysters (selected and wild-type) to the parasite *Bonamia ostreae* at the cellular and molecular level.

Experimental infections by injection of purified parasites have been widely used to study interactions between parasite and host and to develop selective breeding programs for resistance to bonamiosis. This method usually induces a high percentage of mortality associated with high levels of parasite detection (Tigé and Grizel, 1984; Elston et al., 1986; Mialhe et al., 1988; Naciri-Graven et al., 1998). Moreover, infection by injection allows subject oysters to a similar infection pressure, as they are inoculated with a similar defined amount of parasites. Despite these advantages, *in vivo* trial does not mimic the natural parasite entry pathway into the oysters.

In our study, we also carried out a cohabitation experiment to induce the parasitic disease. For this purpose, we used experimentally infected oysters as the source of contamination of healthy oysters (Lallias et al., 2008). Cohabitation experiment is close to a natural infection but the parasite is only detected after 3 months of challenge and challenged oysters usually display higher heterogeneity of infection level (Lallias et al., 2008).

In the present study, selected flat oysters displayed lower mortality rates and parasite detection frequency than wild-type oysters. Observations of histological sections showed a wide distribution of the parasite in different tissues (including digestive gland, gills, mantle and gonad) in infected wild oysters. Infection was sometimes associated with haemocyte infiltration in wild-type oysters. On the contrary, in infected selected oysters, the infection appeared less spread out and the parasite was mainly observed in gills and digestive gland.

The dynamic of measured haemocyte activities in parasite injected oysters was different between wild and selected oysters. The latter showed a decrease of phagocytosis capacity followed by a decrease of ROS production. In contrast, wild oysters generally showed an increase of the different measured activities, first of ROS production, then phagocytosis capacity and lastly esterase activities.

Moreover, fluorescent bead phagocytosis was lower in infected selected oysters compared to non-infected ones whatever the type of experiment. No significant difference was observed between infected and non-infected wild type oysters except 15 days post-injection: infected oysters showed higher phagocytosis activity than controls. In a previous work, a decrease of phagocytosis was also observed 2 h after *in vitro* infection (Morga et al., 2011a). Although phagocytosis may allow parasite degradation, it also contributes to the establishment and spread of *Bonamia ostreae* infection in target cells, the haemocytes. By decreasing phagocytosis activity, selected oysters might be able to reduce disease development. Wild infected oysters tended to increase their haemocyte activities while the selected infected oysters seemed to decrease their phagocytosis capacity, which could be a way to avoid the spreading of the parasite. In cohabitation experiment, once the infection was well established, selected infected oysters maintained a lower level of phagocytosis capacity than selected non-infected oysters.

Non-specific esterases activities did not show significant differences between tested conditions except in wild-type oysters 30 days post-injection. Infected oysters showed higher esterase activities than controls, which could be related to the development of the infection and the activation of the host response.

Tested conditions did not affect ROS production during the cohabitation experiment whereas in the context of the injection experiment, differences were observed between infected and non-infected oysters. Infected wild-type oysters produced more ROS 3 and 5 days post-injection, while infected selected oysters produced less ROS, especially 8 days and 30 days post-infection. Reduced ROS production could be due to a decrease of the cellular damage. In contrast, in infected wild-type oysters, an important production of ROS was observed. This increase in ROS production may be linked to a higher presence of the parasite compared to selected oysters as shown by histological observation.

Expression levels of four genes were monitored during the experimental infections. These genes were previously identified in the context of *in vitro* infections (Morga et al., 2010, 2011a,b, 2012).

Two genes are related to the respiratory burst and antioxidant response (OeSOD and OeEc-SOD) and the other two genes belong to the apoptosis pathway (Oe-Fas ligand and OeIAP). SOD enzymes (cytoplasmic or extracellular) are known to be involved in the oxidative stress response through ${\text{O}}_{2}^{-}$.detoxification. ${\text{O}}_{2}^{-}$ is toxic for pathogen agents and haemocytes as well.

FSW injected selected or wild oysters did not show significant modulation in time.

In selected oysters injected with the parasite, the first significant observed modulation was an increased expression of OeFas-ligand at 3 and 5 days post-injection, followed by an up regulation of OeIAP and OeSOD after 5 days. Oe-EcSOD expression appeared significantly down-regulated after 8 days post-infection. The wild oysters injected with the parasite displayed more diverse responses but did not show an increase of expression of genes related to apoptosis. On the contrary these genes as well as the OeSOD appeared under expressed at 3 and 5 days. The observed over-expression of OeFas-ligand 3 days post-injection in selected infected oysters may indicate that resistance to bonamiosis partly relies on apoptosis, which contributes to destroy infected cells. The expression of the OeEc-SOD was generally decreased during the experiment in the selected infected oysters.

In wild infected oysters, the parasite seems to inhibit OeIAP and OeSOD expression possibly to avoid its own degradation by haemocytes. The inhibition of oxygen radical production has already been described in other host-parasite models and facilitates intracellular survival of protozoan parasites including *Trypanosoma* spp. (Penketh et al., 1987), *Toxoplasma* spp. (Murray et al., 1980; Shrestha et al., 2006), *Leishmania* spp. (Murray, 1981), *B. ostreae* (Morga et al., 2009) and *P. marinus* (Volety and Chu, 1995).

In the cohabitation experiment, both wild and selected infected oysters showed an increased expression of OeFas-ligand suggesting that the stimulation of apoptosis is a natural response of the oyster against the parasite. On the contrary, the decrease of OeEc-SOD was only observed in selected infected oyster and may be related to the decrease of phagocytosis observed in these oysters.

Although injection experiments allow to describe the development of the infection, this experimental approach does not take into consideration the whole pathogenesis process. On the contrary, cohabitation experiments allow to investigate all the interactions between parasites and the host, including the potential role of barriers like mucus or mantle in the response of the oysters to the disease. *Perkinsus marinus* is an important protistan parasite of the eastern oyster *Crassostrea virginica*. Recent findings showed that oyster pallial organs (mantle, gills) are a major portal of entry for the parasite Pales Espinosa et al., 2013, 2014).

Selected and wild oysters in contact with infected oysters showed differences of mortality rate as well as differences of prevalence and infection level. However, compared to the injection experiment, the response of the oysters appeared to be more variable and delayed in time.

These experimental challenges contributed to investigation the response of selected and wild oysters during the course of an infection through the injection of parasites and through cohabitation with infected oysters. We observe a difference of the response according the two flat oyster groups tested however the difference is not always clear. This small difference could be due to the use of flat oyster from Quiberon; Flat oysters are long-exposed, and may have been selected to some extent. If the wild-type population has not been exposure to *B. ostreae*, the difference between both populations is stronger.

As soon as 3 and 5 days post-infection, infected selected oysters are able to decrease their phagocytosis capacity and increase the transcription of genes related to the apoptosis. These results could explain the lower level of infection and prevalence observed in these oysters. Decrease of phagocytosis activity and modulation of apoptosis induced by the parasite and/or by the oyster appear as two of the key mechanisms supporting resistance to bonamiosis. The parasite seems to be more able to infect flat oyster than cupped oyster haemocytes and the apoptotic response was more important against live than dead parasites in the natural host than in *C. gigas*. These results suggest that *O. edulis* specifically responds to *B. ostreae* by inducing apoptosis of haemocytes. These results give new perspectives in the understanding and the management of the disease and might contribute to identify new target for the development of selection programme.

## Author contributions

BM, TR, NF, SoL, CG, BC, JJ, SyL EH, and IA contributed to the conception of the work. BM, TR, NF, SoL, CG, BC, and JJ acquired and analyzed the data for the work. BM, TR, and IA interpreted the data. BM, TR, and IA drafted the work.

### Conflict of interest statement

The authors declare that the research was conducted in the absence of any commercial or financial relationships that could be construed as a potential conflict of interest.
